# Improving the way healthcare professionals deliver different news to families during pregnancy or at birth: a qualitative study

**DOI:** 10.1017/S1463423620000651

**Published:** 2021-03-29

**Authors:** Esther Mugweni, Samantha Goodliffe, Sabrena Jaswal, Melita Walker, Angela Emrys-Jones, Cheryll Adams, Sally Kendall

**Affiliations:** 1 Institute of Health Visiting c/o Royal Society for Public Health, London, UK; 2 Cornwall Down Syndrome Support Group, Cornwall, UK; 3 Centre for Health Services Studies, University of Kent, Kent, UK

**Keywords:** communication skills, congenital anomalies, difficult news, learning disability

## Abstract

**Aim:**

To explore the lived experience of delivering or receiving news about an unborn or newborn child having a condition associated with a learning disability in order to inform the development of a training intervention for healthcare professionals. We refer to this news as different news.

**Background:**

How healthcare professionals deliver different news to parents affects the way they adjust to the situation, the wellbeing of their child and their ongoing engagement with services. This is the first study that examined the lived experience of delivering and receiving different news, in order to inform the development of training for healthcare professionals using the Theoretical Domains Framework version 2.

**Method:**

We conducted qualitative interviews with a purposive sample of 9 different parents with the lived experience of receiving different news and 12 healthcare professionals who delivered different news. It was through these descriptions of the lived experience that barriers and facilitators to effectively delivering different news were identified to inform the training programme. Data analysis was guided by Theoretical Domains Framework version 2 to identify these barriers and facilitators as well as the content of a training intervention.

**Findings:**

Receiving different news had a significant impact on parents’ emotional and mental wellbeing. They remembered how professionals described their child, the quality of care and emotional support they received. The process had a significant impact on the parent–child relationship and the relationship between the family and healthcare professionals.

Delivering different news was challenging for some healthcare professionals due to lack of training. Future training informed by parents’ experiences should equip professionals to demonstrate empathy, compassion, provide a balanced description of conditions and make referrals for further care and support. This can minimise the negative psychological impact of the news, maximise psychological wellbeing of families and reduce the burden on primary care services.

## Introduction

As part of the NHS Fetal Anomaly Screening Programme, eligible pregnant women in the UK are offered screening to assess the probability of their baby being born with Down’s Syndrome, Edwards’ Syndrome, Patau’s syndrome and eight other structural abnormalities (PHE, 2017). Some congenital anomalies such as Down’s Syndrome may be associated with a learning disability. Identifying women with an increased chance of having a child with fetal anomalies, enables healthcare professionals (HCPs) and the families to discuss definitive prenatal diagnostic tests; treatment options if available; appropriate plans for delivery and have discussions about whether or not to continue the pregnancy (PHE, 2017). While antenatal screening may identify some anomalies during pregnancy, some are not identified until after birth.

When anomalies are identified, families are faced with the unexpected experience of receiving different news about their unborn or newly born child. The term ‘different news’ was used in this study to describe the process of receiving information relating to an unborn or newly born child being diagnosed with a condition associated with a learning disability. We used the term based on feedback from parent representatives who were part of the study team and who preferred this term instead of bad news for two reasons. Firstly, the term bad news gives a negative connotation about the child and diagnosis to families and HCPs. Secondly, different news was thought to more accurately reflect the idea that news about the diagnosis is different from what parents expected about their child but not necessarily bad.

How HCPs deliver different news is an important factor in how it is received, interpreted, understood and processed by parents (RCN, 2013). A literature review exploring the effects of prenatal diagnosis found that parents experience a range of negative emotions immediately after receiving different news including fear, worry, anxiety, helplessness, anger, hopelessness and sadness (Luz *et al.*, 2017). Studies have shown a clear correlation between maternal anxiety and the focus of the initial conversation when HCPs delivered different news thus highlighting the importance of how it is delivered (Skotko *et al.*, 2009; Fonseca *et al.*, 2014). Some parents go on to experience chronic stress, depression, anxiety or other mental health conditions which may affect fetal programming and post-birth impact on the social-emotional, cognitive and physical development of children (Deater-Deckard, 2005). Chronic stress and parental mental illness are known risk factors for poorer health outcomes across the child’s life course (Stein *et al.*, 2014).

This has significant implications for primary care in particular for General Practitioners (GPs) and Health Visiting services who are often the first point of contact for the treatment of perinatal mental health problems for parents (Bauer *et al.*, 2016). Primary care services also have to provide higher levels of support to mitigate any problems arising from the parent-infant relationship to improve outcomes for families (Morrell *et al.*, 2009). A cross-sectional survey of parents found that over a period of six months, parents who were well supported and had access to appropriate information when they needed it, adjusted well to the diagnosis; showed patterns of resilience; had reduced symptoms of anxiety and depression and improved quality of life (Fonseca *et al.*, 2012).

Effective delivery of different news requires excellent verbal and non-verbal communication skills (RCN, 2013). However, there is a recognition that this process may be difficult and stressful for HCPs (Wolfe *et al.*, 2014; Luz *et al.*, 2017). Many HCPs report feeling unprepared for delivering different news to parents (Luz *et al.*, 2017). Often HCPs have learnt to deliver different news from the ‘see-one-do-one’ approach. This is limited because of the variation in the skills of the senior HCPs observed by junior colleagues (Kim *et al.*, 2016; Atienza-Carrasco *et al.*, 2018). Simulations, reflective practice, debriefing and lectures have been used to teach HCPs how to deliver different news in the paediatrics and obstetric settings (Karkowsky *et al.*, 2016; Kim *et al.*, 2016). A limitation of these training interventions is many have not been trialled in the UK in conjunction with minimal evidence of the transfer of skills into practice. Additionally, the delivery of different news in some of these studies included oncology (Kersun *et al.*, 2009) for example, where parents will be anticipating difficult news which may not be the case in maternity care, and different news is unexpected for both the parents and HCPs.

The literature demonstrates the need for an evidence-based training programme to address the needs of both parents and HCPs. Previous research on parents lived experiences of different news related to a learning disability in the UK is scarce. To the best of our knowledge, only one previous study has been conducted in the UK exploring mothers lived experiences of being told of a fetal anomaly (Lalor *et al.*, 2007). This study did not look at subsequent development of a training intervention. Given the substantial impact that different news can have on families and the lack of standardised training for this role, it is imperative that HCPs are provided with training to enable them to maximise psychological wellbeing of the whole family through effective support.

This study aimed to explore the lived experience of delivering or receiving news about an unborn or newborn child having a condition associated with a learning disability in order to inform the development of a training intervention for HCPs. The study examined what was working well in this area, the gaps in practice as well as support and training needs, in order to use the study findings to develop a corresponding training intervention that corresponds with parents’ needs and experiences.

## Method

We conducted a qualitative study using in-depth interviews in May 2018 in the UK (Appendix 3). In-depth interviews were selected due to the sensitive nature of the topic. We received ethics approval to conduct this study from the Cambridge East Research Ethics Committee (REC Reference 17/EE/0410).

We used purposive sampling to recruit parents from different families with the lived experience of receiving different news about their unborn or newborn. Specifically, we used intensity sampling as we recruited parents who had the first-hand experience of receiving different news. Parents were recruited from various charities using study flyers or the participant information sheets distributed during usual meetings, appointments or communication updates by representatives from the charities or by the HCP. Parents who were interested in the study contacted the study team directly by telephone and/or email and those who met the study criteria (i.e. families who had the lived experience of receiving different news) and consented, were enrolled in the study.

We also used intensity sampling to recruit HCPs who delivered different news from NHS Trusts supported by Health Education England (HEE) working across Kent, Surrey and Sussex. Email invitations to participate in the interviews were sent out via HEE networks (such as Heads of Midwifery, Paediatrics, Obstetrics, Ultrasound, Neonatology) to pass on to all eligible staff members. Interested participants contacted the research team directly and those who fulfilled the inclusion criteria (that is were regularly involved in the delivery of different news) and consented were recruited into the study. This intensity sampling approach was adopted due to the sensitivity of the topic; we recognize that this small sample is not a maximum variation sample of all parents receiving different news or all fetal anomaly conditions. However, the sample generated rich, in depth data.

Two interview guides were used for data collection, one for parents and the other for HCPs (Appendix 1 and Appendix 2). The interview guides for the families and HCPs were developed based on the literature review undertaken at the start of the study which has been published elsewhere (Mugweni *et al.*, 2019). The literature review aimed to provide the context in which families receiving different news in the UK; examine the impact of this news on families; clarify good practice when delivering different news and identify areas for further training and development for HCPs who deliver different news.

In addition to the literature review, a consultation was conducted with parent representatives on the content of the interview guides. The interviews examined the process of receiving or delivering different news, what went well within that process and what could have been improved upon. For example, parents were asked *what were your thoughts about the healthcare professional who gave you the news?* This was then followed by two sub-questions asking parents what they felt the HCP did and did not do well in the delivery of this different news. Similarly, HCPs were asked to recount a time when they delivered different news and then elaborate on what they thought went well about that particular experience and what they felt they needed to improve upon.

Parents and HCPs also identified perceived training needs and possible interventions to address the identified challenges. For example, *what skills or training do you think healthcare professionals who deliver news need to have?*

Interviews with HCPs were conducted over the phone while interviews with parents/families were conducted face-to-face at a mutually convenient time and place. Telephone interviews were used due to the need to be flexible around the busy schedules of clinicians. This meant that we did not capture some of the body language, such as facial expressions, but other non-verbal language such as tone of voice was captured. Telephone interviews are used extensively in health services research and were a valid method for this study. Both HCPs and family interviews lasted between 45 min and 1 h. Interviews were audio-recorded and transcribed verbatim and anonymised before analysis. The interviews were undertaken by EM and SG; two female researchers with a PhD and MSc respectively with no affiliations to the study sites or participants prior to the interviews. Field notes were made by EM and SG during data collection. Interviewer bias was reduced by involving the whole team in the development of the interview guide and cross-checking data interpretations.

### Analysis

All qualitative data were managed using NVivo and analysed using Framework analysis (Ritchie and Spencer, 1994). The analysis of the interviews was undertaken by EM and SJ. Given that the findings from the study were to inform the development of a training intervention aimed at changing clinical practice, we drew on the Theoretical Domains Framework (TDF) version 2 in the analysis of the lived experiences (Cane *et al.*, 2012; Atkins *et al.*, 2017). The TDF is a flexible evidence-based framework aimed to help apply theoretical approaches to interventions aimed at behaviour change. The TDF framework is particularly suited to mitigating the complexity inherent in developing interventions aimed at changing clinical practice/behaviour and has been used extensively for this purpose for example (Murphy *et al.*, 2014; Mangurian *et al.*, 2017; Chapman *et al.*, 2018). The TDF has 14 domains providing detailed information to identify determinants of behaviour (TDF) these as shown in Table 1. Mapping barriers and facilitators to change in practice onto the TDF can then be followed by the identification of appropriate intervention functions and behaviour techniques from the behaviour change technique taxonomy (Michie *et al.*, 2013).

**Table 1.**
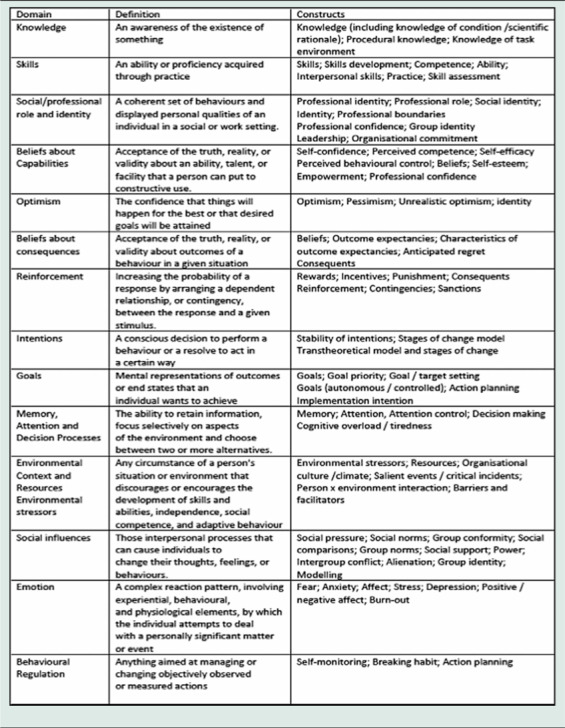
Definitions Of domains of version 2 of the theoretical domains framework (Cane *et al.*, 2012)

In this study, we used the TDF version 2 to identify barriers and facilitators to effectively delivering different news to determine what would need to be included in a future training intervention for HCPs. The analysis aimed to highlight factors that were perceived as important in delivering different news sensitively based on parental experience data. Only those domains which were relevant to the study and which emerged in the data were included in the analysis. We began Framework analysis with familiarisation with the data, followed by the development of a thematic framework to index the data (Ritchie and Spencer, 1994). This was followed by charting the data then mapping and interpretation of data through descriptive and explanatory accounts (Ritchie and Spencer, 1994). Findings were illustrated using anonymised quotations and pseudonyms.

## Results

### Description of participants

Twelve HCPs participated in the interviews namely: two consultants in obstetrics and gynaecology; one neonatologist; eight midwives (including screening midwives and fetal medicine midwives and one registrar in obstetrics and gynaecology). Nine parents of different children were recruited: six had children with Down’s Syndrome and three who had children with other rare chromosol disorders. Six parents received the news at birth, two received the news during the first-trimester screening and one at 34 weeks of pregnancy during a growth scan. Six of the parents indicated that they took career breaks to look after their children. Whilst the sample could not represent all fetal anomaly conditions, it was sufficient to capture the experience of receiving and delivering different news to allow data saturation in terms of scope and replicability (Morse, 2015).

### Factors to consider when delivering different news

#### Professional roles

This domain referred to the professional roles and boundaries of HCPs involved in delivering different news. Care for mothers during pregnancy and after birth was provided by multidisciplinary teams. Sonographers identified concerns during ultrasound screening; however, the consultant was responsible for discussing the findings with the family:

*So, she’s (sonographer), like “Oh, I need to go and get a doctor”, and there was a shadow, a black shadow, that’s all there was…”* (Parent 3)

*She (sonographer) acted as usual and she didn’t react, you know, like, oh, anything like that, but at the end, she said, “I saw something that’s not quite right, but I can’t say anything, so I have to speak to the…to speak the doctor” So, she went out…and then she came back ….and then she said, “Yes if you can come back at one o’clock.”* (Parent 4)

Midwives identified concerns as part of the early baby check or after they received the results of the combined screening test from the laboratory. Screening midwives delivered results over the phone, followed by an email to parents who would subsequently be invited to meet with the respective midwife at their earliest convenience to discuss the results; receive a referral to other relevant departments such as fetal medicine or to arrange for confirmatory tests. At birth, it was the consultant’s responsibility to establish the diagnosis, communicate this to parents and answer their questions:

*You can’t say, yes, your baby has Down’s until the chromosomal test is back and that’s quite difficult because the parents want a definite and you can only say it’s suspicious of and that sort of thing and I personally found that quite difficult.* (HCP 1)

*You have to prepare before you see the patient, so you’re able to answer their questions about like what’s the meaning of chromosomes, or like … what investigations will they have to have or what complications, what does it mean?* (HCP 3)

In terms of professional roles, the data suggested that future training needs to target all members of the multidisciplinary team providing care to the families. It would be important to also identify ways to improve each aspect of the process from the time that concerns are raised to the time that an official diagnosis is provided by recognising that these are not discrete events but are all part of the process of delivering different news.

#### Knowledge, skills and beliefs about capabilities

This domain referred to the perceived knowledge and skills to deliver different news. Experience and competence were described as factors that either enabled or hindered the effective delivery of different news by HCPs.

A. Experience

Lack of experience was perceived as a major barrier to effectively conveying different news by HCPs:

*The more opportunities you have to break news like this, I think the more comfortable you become at changing your approach during the consultation, as a junior… it’s very easy to become tongue-tied.* (HCP 10)

*Reading the triggers, reading, because so much of it focusses on sort of watching and listening and observing, and just kind of picking up on cues which I guess only comes with experience.* (HCP 12)

The above discourse suggests that the more experience an HCP had at delivering different news the more likely it was that they improved their technique. However, some experienced clinicians were described as needing to improve their technique:

*I have two consultants that I work with….one of them is incredibly proficient at it….The other consultant…isn’t as proficient at it.* (HCP 5)

B. Competence

HCPs often indicated that they had received communication training with a sub-component on difficult news as part of their undergraduate or postgraduate training. Only one HCP reported attending a specific different news course. For most, acquisition of knowledge and skills on delivering different news was based on observing more senior colleagues. Although this is a common form of learning in clinical practice, it had limitations if the senior clinician was not perceived as being very good at delivering different news.

No matter how competence was acquired, all participants felt that it was important for HCPs who deliver different news to be adequately trained to do this sensitively and to also be able to provide effective support to parents and/or referral to appropriate support services. All study participants also felt that HCPs needed to be compassionate and empathetic:

*You could tell that he, he really cared about our little boy…and he said that “your lovely little boy is just going to need some extra help” and um he was just really genuinely caring and, very kind in how he put the news.* (Parent 7)

*They said it as if they cared…they were sensitive. They said it as if this person in front of them was their best friend.* (HCP 2)

Compassion was also important because of the immediate psychological and emotional impact of receiving different news on parents. Parents reported feeling shocked, guilty, ashamed and deep sorrow:

*xxxx (husband) and me didn’t really respond very much to each other for a long time, even talk to each other. I had very severe postnatal depression.* (Parent 3)

*My husband went to see my parents and told them and just felt awful, he was like, “oh, I felt bad and I felt really guilty telling them this.”* (Parent 5)

Participants also highlighted the importance of the HCP being tactful in their choice of words as well as their non-verbal language that could be misinterpreted for blaming parents or making the birth of their children regrettable:

*She had a very small baby with Down’s Syndrome and so they had come to talk about further screening and I said, “oh, you know, if you want another test to find out the risk of this happening again”, and then, of course, that was it, the consultation just pretty much ended there when the grandmother said, “Well, why are you saying the risk of this happening again? We wouldn’t mind if it happened again, this would be absolutely fine”…and, everything broke down at that point, I had to go and get someone else in because I’d said the wrong words.* (HCP 12)

The above accounts highlight the importance of HCPs having a non-judgemental, caring, compassionate attitude to minimise the negative impact and to enable parental confidence to accept and access ongoing support. Only one parent reported immediate acceptance after the news was delivered to her because she had known someone with a similar condition to their child who had done reasonably well in terms of their health, development and family life. Parents suggested that future training could benefit from including the lived experience of receiving different news so that HCPs developed an understanding of the impact of how different news is delivered as well as insight into the general impact of the news on a family.

#### Environmental Context and Resources

Privacy and time were highlighted as important factors for effectively delivering different news.

A. Privacy

Some HCP’s noted that their hospitals did not have the physical space to accommodate the delivery of different news privately:

*It’s, unfortunately, it’s not ideal cause we are really, we haven’t got a lot of space on our unit, but unfortunately, it all has to be done within the unit where there’s still work going on.* (HCP 6)

*I think maybe, if we’d been in a private room away from the kind of being in the intensive care unit that might have helped in some respects because we would have been able to kind of you know, cry or talk about it but because we were like*

*…in NICU, I felt I had to hold my emotions in, that I couldn’t kind of just…If, if I could go back and do it again, I would have asked that we could have been moved, or we could have been in a room where we could have been left to just, to process ourselves rather than being in the intensive care unit.* (Parent 6)

However, several participants reported that after the news was delivered postnatally, parents were often looked after in the bereavement suite. Parents expressed mixed feelings about this. While in some instances this gave them the privacy to process their own emotions, being in a bereavement suite also reinforced the negative connotations of the birth of their child:

*It’s a bit of a double-edged sword. We were moved into a room by our self, which was good because me and my husband literally as soon as we got in that room we just cried and hugged each other…It did feel a little bit like we’d been…shoved right out of the way as well.* (Parent 6)

B. Time

HCPs often had a significant number of patients they were looking after which sometimes hindered a patient-centered approach to delivering different news:

*There are other babies which need delivery as well…so that is very challenging, and you just apologise to the patient …. “I am very sorry, I have to, go for an emergency, and will come back.”* (HCP 4)

Several parents indicated that it was important for the HCPs to have time to answer questions after the diagnosis; however, this was not always possible in busy wards. It was also important to consider the time of day at which the HCP decided to deliver the news:

*I did feel that the doctor who delivered the news at XXX Hospital…I felt that was done very sensitively and I was, I was happy with the way they did that, although I would have preferred that my husband was with me at the time and that I wasn’t on my own [laughs] in the hospital having just given birth, feeling a bit isolated.* (Parent 9)

Some parents reported that they would have preferred that the HCPs had delivered the news when they were not tired or alone. In another example, the HCP did not take into consideration both the need for a private space or the appropriateness of delivering different news within minutes of the child’s birth:

*…maybe if she’d have waited a bit or gone out of the room and thought about it before she said what she said…And just given us 20 minutes or something…But it was literally, if you can imagine, so you’ve got xxxx [the baby] lifted up, shown she’s a girl, said, “Do you want to tell your husband what it is?” so they then carry her off and I go, “It’s a girl xxxx, it’s a girl,” he burst into tears, happiness, and then I look around for someone’s trying to get my attention, and she’s like, “Oh, you know, your baby’s got all its fingers and toes but I think she’s got Down’s Syndrome,” and it’s like it all happened, just like, and…[she’s] over there now with other people and I’m like, and there’s just all these people looking at me that they’re gonna get ready to stitch me up, and, you know…So, I’m still sort of like looking at everybody looking me, waiting for me to…I don’t know, do whatever they want me to do.* (Parent 8)

Parent 8 made the point that the news was given abruptly and that the there was no need to tell her life-changing information within minutes of her baby being born, in the presence of many other HCPs and most importantly before she had been given the opportunity to hold her baby for the first time, celebrate her birth, feed and bond with her daughter. In a contrary scenario, the consultant noted some markers for Down’s Syndrome; however, they dealt with the medical emergencies first and raised their concerns when the baby was three days old:

*She was obviously kind of putting the pieces together …. and so she had her kind of concerns that, that xxxx [the baby] was born with Down’s syndrome and it hadn’t kind of been voiced, but I mean it was only three days into him being here, bless him, so, um, so she kind of, she said to us about it and said “I’d like to do a blood test…”* (Parent 7)

It is important to note that medical concerns for the mum or baby created scenarios in which communication was abrupt or non-existent from HCP’s in an attempt to deal with the emergency at hand. However, where there were no immediate life-threatening emergencies for the mother or the baby, parents felt that it was important for the HCP to take time to prepare themselves to deliver the news by finding a private place to talk to parent; getting up to date information about the diagnosis as well as long- and short-term support available to parents. It was important to take time to consider the most appropriate time of the day to deliver the news, bearing in mind the importance of including significant others such as spouses and to have time to answer questions or identifying someone to take over if they knew that they would potentially be required to attend to other parents. Assessing the physical and social environment before delivering different news was perceived as a critical component to minimising the negative impact of receiving different news.

#### Optimism

Optimism referred to HCPs being able to reassure parents by providing a balanced description of their child, so the parents are able to make an informed decision about continuing or terminating pregnancy as is indicated below:

*We do need to be careful what we say, we do need to get that balance right, we do need to support the women in whichever choice…* (HCP 2)

*They were very, you know, “We recommend you terminate”, not as in, “If she survives, she may have these problems”, there wasn’t any of that, it was, “We recommend you terminate and this is what happens”, rather than, you know, “If she survives she may not be able to do this, this, this and this”, it wasn’t a balanced conversation.* (Parent 3)

While HCPs felt that it was important to be able to provide parents with information about the condition their child had been diagnosed with, this needed to be given in stages as some parents mentioned being overwhelmed and unable to take in the large amount of information provided to them. It was also important to parents that HCPs provided a balanced description of their child’s diagnosis and its implications both in the short- and long-term future without an overemphasis of negative things which may or may not happen in the future such as developing Alzheimer’s disease. Parents felt it was unfair to discuss such issues for children with Down’s Syndrome for example and leave the discussion of similar matters with parents of neurotypical children who could well have a family history of similar diseases and potentially have a genetic predisposition to this. Parents with lived experience stressed the need for the training intervention to highlight some of the challenges as well as the joys of having a child who was diagnosed with a congenital anomaly.

#### Emotion

Several HCPs indicated that delivering different news on an ongoing basis affected them emotionally and that sometimes the effect of the conversations lingered for years after the event:

*You think “oh my God if that was my baby how would I feel"…how would I feel if that was me or that was my daughter or my sister or my mother?* (HCP 3)

*I have gone into that woman’s world completely unannounced and I’ve destroyed it…I have walked out of a feticide and absolutely broken my heart…but it’s part of the job, isn’t it?* (HCP 2)

*It affects me, of course, …but I have to accept that this is my job* (HCP 4)

While some NHS trusts were said to have facilities in place for team debriefing, this was not a universal experience. HCPs indicated that future training would need to support HCPs to identify ways to acknowledge and manage their own emotions about delivering different news to enable them to build emotional resilience so that they could provide parents with good care.

## Discussion

To the best of our knowledge, this is the first study that examined the lived experience of receiving or delivering different news in order to develop training for HCPs using the TDF version 2. Based on parental experience and HCPs perceptions, our study identified barriers and facilitators to the delivery of different news, the impact of the news as well as the perceived training needs for HCPs. The findings indicate that how different news was delivered had a significant impact upon parent’s emotional and mental wellbeing, the parent–child relationship, the parents’ relationship with one another and the relationship between the family and the HCPs as highlighted in previous studies (Dent and Carey, 2006; Sheets *et al.*, 2012; Edvardsson *et al.*, 2018).

These findings suggest that it is crucial to ensure that parents who receive different news have access to an HCP with the skills to deliver the news sensitively and compassionately. Our study suggests that not every family has access to such HCPs when they need them due to lack of standardised training on how to effectively deliver different news as highlighted by other authors, pressure on the wards and system failures (RCN, 2013, Luz *et al.*, 2017). However, providing evidence-based communication skills training that reflects the needs of the families with lived experience is consistent with the NHS Long-Term Plan to improve understanding of the needs of people with learning disabilities and their families and to work with them to improve their health, wellbeing and access to timely support (NHS, 2019). Such training could reduce the psychological impact on parents and therefore any associated costs and burden of perinatal and infant mental health problems on primary care services, which forms a crucial part of ongoing support service for parents after diagnosis.

Parents and HCPs suggested that future training needs to equip HCPs to; demonstrate empathy, show compassion, be flexible with time or plan around the demands of their ward, utilise kind, simple and truthful language; provide a balanced description of the condition; offer sufficient time to answer questions and make appropriate referrals for further care and support. Some of these key aspects of delivering different news have also been highlighted in other works (Dent and Carey, 2006; Skotko *et al.*, 2009; Sheets *et al.*, 2012; Luz *et al.*, 2017; Edvardsson *et al.*, 2018).

The training approach is relevant across all areas of health services maternity provision, including primary care. For many parents receiving different news in the antenatal period or for their new-born and having on-going support from their GP, community midwife, practice nurse and health visitor will be essential. The communication between the hospital maternity services, diagnostics and primary care must be sufficient to ensure parents are not retraumatised by having to repeat their story. Providing continuity of care between primary and secondary care services is integral to this. As gatekeepers in the UK primary care system, GPs and nurses could be pivotal in ensuring integration between hospital and community care provision (Valentijn *et al.*, 2015).

Our follow-on study will coordinate findings from previous studies and from this study to develop a training intervention for delivering different news, which has not been reported in previous studies. Health Education England needs to use the findings from this study and any follow-on studies on delivering different news training to close the gap between research and practice by developing appropriate policy to ensure that the key principles of this delivery of different news training becomes part of core modules during undergraduate and postgraduate education as well as mandatory training for continuous professional development to ensure consistent safe and balanced practice.

### Strengths and limitations

We used qualitative in-depth interviews to explore the lived experiences of receiving or delivering different news to inform our understanding of the barriers and facilitators that could be addressed in a training programme for HCPs. The interviews produced a significant amount of data allowing the team to develop a thick description of lived experiences from very hard-to-reach families so that the future training can be underpinned by the real-life experiences of families. Another strength of the study is the use of TDF in the analysis of the study findings to support intervention development. Use of theory to understand the mechanisms of action of intervention strategies has been shown to improve their effectiveness (Michie *et al.*, 2011).

Whilst our purposive samples were relatively small, the findings from this study will be potentially transferable to similar populations as we drew on a range of different parents and were also able to reach data saturation in terms of scope and replicability. Findings are therefore potentially transferable to the delivery of different news in other settings, although further research and testing of the training would provide further evidence. While the diversity of the HCP group enabled the team to obtain varied experiences on delivering different news, future studies could also look at finding innovative ways to recruit families with other different conditions as well as incorporating the experiences of those who choose to terminate a pregnancy based on fetal anomaly screening.

### Implications for future research and practice

A crucial next step of the study will be the development of a training intervention. The appropriate intervention functions and behaviour techniques can be identified using the behaviour change technique taxonomy (Michie *et al.*, 2013). It will be necessary to assess the acceptability and feasibility of implementing the training intervention to HCPs. Following on from the feasibility study we would recommend a definitive large-scale trial to look at the implementation of the training in different settings and its impact on family outcomes as well as the cost-effectiveness of the intervention.

## Appendix 1 Parent Interview Guide

Improving the **D**elivery of **D**ifferent **N**ews to families by healthcare professionals.

### Demographics

1. Can you tell me a little bit about yourself:
  a. Age? What you like to do during your spare time? What you do for a living? Whether or not you have a partner? How many other children do you have? – do they have a learning disability?

### Different news

2. Could you please tell me about when it was first suggested that your child has a learning disability?
3. What did the suggestion of a learning disability mean to you?
  a. Has your child had a formal diagnosis?
4. How was the news delivered and by whom?
  a. Where were you told?
  b. Who was with you?
  c. Were you given any information?
  d. Were you given enough time?
5. How did the way the news was delivered to you make you feel?
6. How did you feel after you had been given the news when the HCP had left?
7. What were your thoughts about the healthcare professional who gave you the news?
  a. In your opinion, what did the HCP do well?
  b. In your opinion, what could have been done better?
8. What do you think should be included in a balanced description of a learning disability such as the one your child was diagnosed with?

### Support

9. How have you managed the diagnosis?
10. What support did you receive after the diagnosis from healthcare professionals?
11. What support do you think should be offered to other parents who go through this experience in the future?

### Training needs

12. What skills or training do you think healthcare professionals who deliver different news need to have?
13. Why are these skills or the training important?

### Conclusion

- Is there anything you would like to add or share which I may have not asked?

## Appendix 2. Health Care Professional Interview Guide

Improving the **D**elivery of **D**ifferent **N**ews to families by healthcare professionals.

### Demographics

1. Can you tell me a little bit about yourself?
  a. What you like to do during your spare time?
  b. Do you have children?
  c. Your profession and the length of time you have been practicing?

### Different news

2. Could you tell me about the protocol for your NHS trust on delivering different news to patients? If yes – how do you find this? If no – what do you think to a protocol?
3. Can you tell about what training you have received in patient communication?
  a. Type of training – did it include role play?
  b. Specific about different news?
  c. When was the training?
  d. How often you deliver different news to families?
4. Can you tell me about a time when you had to break different news to a family?
  a. Do you remember what went well?
  b. What did not go as well as you had planned?
  c. Could you give me an example of how you tell parents?
5. How did the process of breaking different news make you feel?
6. In your opinion what are the factors which can make the process of delivering different news go well?
7. From your own experience or that of other health professionals you know, what can make the process challenging?
8. What do you think makes a balanced informing interview about a child having a learning disability either during pregnancy or at birth?

### Support

9. What support does your Trust offer families after they receive different news?
10. What support do you think should be offered to other parents who go through this experience in the future?
11. What support does your Trust offer health professionals who deliver different news?
  a. Do you think health professionals who deliver different news need support?
  b. If yes what would this look like?
  c. If no, why not?

### Training needs

12. What skills or training do you think healthcare professionals who deliver different news need to have?
13. Why are these skills or the training important?

### Conclusion

- Is there anything you would like to add or share which I may have not asked?
- Would you like to ask me anything?

Thank you for sharing your experience and thoughts with me and for your time today.

## Appendix 3 COREQ (Consolidated criteria for Reporting Qualitative research) Checklist

**Table:**

Topic	Item No.	Guide Questions/Description	Reported on Page No.
**Domain 1: Research team and reﬂexivity**			
*Personal characteristics*			
Interviewer/facilitator	1	Which author/s conducted the interview or focus group?	*5*
Credentials	2	What were the researcher’s credentials? For example PhD, MD	*5*
Occupation	3	What was their occupation at the time of the study?	5
Experience and training	5	What experience or training did the researcher have?	Title Page
*Relationship with participants*			
Relationship established	6	Was a relationship established prior to study commencement?	*5*
Participant knowledge of the interviewer	7	What did the participants know about the researcher? For example personal goals, reasons for doing the research	N/A
Interviewer characteristics	8	What characteristics were reported about the inter viewer/facilitator? For example Bias, assumptions, reasons and interests in the research topic	*5*
**Domain 2: Study design**			
*Theoretical framework*			
Methodological orientation and Theory	9	What methodological orientation was stated to underpin the study? For example grounded theory, discourse analysis, ethnography, phenomenology, content analysis	*5*
*Participant selection*			
Sampling	10	How were participants selected? For example purposive, convenience, consecutive, snowball	4
Method of approach	11	How were participants approached? For example, face-to-face, telephone, mail, email	4
Sample size	12	How many participants were in the study?	6
Non-participation	13	How many people refused to participate or dropped out? Reasons?	N/A
*Setting*			
Setting of data collection	14	Where was the data collected? For example home, clinic, workplace	*5*
Presence of non-participants	15	Was anyone else present besides the participants and researchers?	N/A
Description of sample	16	What are the important characteristics of the sample? For example demographic data, date	6
*Data collection*			
Interview guide	17	Were questions, prompts, guides provided by the authors? Was it pilot tested?	4
Repeat interviews	18	Were repeat interviews carried out? If yes, how many?	N/A
Audio/visual recording	19	Did the research use audio or visual recording to collect the data?	*5*
Field notes	20	Were ﬁeld notes made during and/or after the interview or focus group?	*5*
Duration	21	What was the duration of the inter views or focus group?	*5*
Data saturation	22	Was data saturation discussed?	6
Transcripts returned	23	Were transcripts returned to participants for comment and/or correction?	N/A
**Domain 3: analysis and ﬁndings**			
*Data analysis*			
Number of data coders	24	How many data coders coded the data?	*5*
Description of the coding tree	25	Did authors provide a description of the coding tree?	5–6
Derivation of themes	26	Were themes identiﬁed in advance or derived from the data?	5–6
Software	27	What software, if applicable, was used to manage the data?	*5*
Participant checking	28	Did participants provide feedback on the ﬁndings?	N/A
*Reporting*			
Quotations presented	29	Were participant quotations presented to illustrate the themes/ﬁndings? Was each quotation identiﬁed? For example participant number	6–13
Data and ﬁndings consistent	30	Was there consistency between the data presented and the ﬁndings?	13
Clarity of major themes	31	Were major themes clearly presented in the ﬁndings?	13–14
Clarity of minor themes	32	Is there a description of diverse cases or discussion of minor themes?	13

Developed from: Tong A, Sainsbury P, Craig J. Consolidated criteria for reporting qualitative research (COREQ): a 32-item checklist for interviews and focus groups. *International Journal for Quality in Health Care*. 2007. Volume 19, Number 6: pp. 349 – 357.
